# Lung-Homing of Endothelial Progenitor Cells and Airway Vascularization Is Only Partially Dependant on Eosinophils in a House Dust Mite-Exposed Mouse Model of Allergic Asthma

**DOI:** 10.1371/journal.pone.0109991

**Published:** 2014-10-03

**Authors:** Nirooya Sivapalan, Jennifer Wattie, Mark D. Inman, Roma Sehmi

**Affiliations:** Asthma Research Group, Firestone Institute for Respiratory Health, St. Joseph's Healthcare, McMaster University, Hamilton, Ontario, Canada; Universidade Federal de Juiz de Fora, Brazil

## Abstract

**Background:**

Asthmatic responses involve a systemic component where activation of the bone marrow leads to mobilization and lung-homing of progenitor cells. This traffic may be driven by stromal cell derived factor-1 (SDF-1), a potent progenitor chemoattractant. We have previously shown that airway angiogenesis, an early remodeling event, can be inhibited by preventing the migration of endothelial progenitor cells (EPC) to the lungs. Given intranasally, AMD3100, a CXCR4 antagonist that inhibits SDF-1 mediated effects, attenuated allergen-induced lung-homing of EPC, vascularization of pulmonary tissue, airway eosinophilia and development of airway hyperresponsiveness. Since SDF-1 is also an eosinophil chemoattractant, we investigated, using a transgenic eosinophil deficient mouse strain (PHIL) whether EPC lung accumulation and lung vascularization in allergic airway responses is dependent on eosinophilic inflammation.

**Methods:**

Wild-type (WT) BALB/c and eosinophil deficient (PHIL) mice were sensitized to house dust mite (HDM) using a chronic exposure protocol and treated with AMD3100 to modulate SDF-1 stimulated progenitor traffic. Following HDM challenge, lung-extracted EPCs were enumerated along with airway inflammation, microvessel density (MVD) and airway methacholine responsiveness (AHR).

**Results:**

Following Ag sensitization, both WT and PHIL mice exhibited HDM-induced increase in airway inflammation, EPC lung-accumulation, lung angiogenesis and AHR. Treatment with AMD3100 significantly attenuated outcome measures in both groups of mice. Significantly lower levels of EPC and a trend for lower vascularization were detected in PHIL versus WT mice.

**Conclusions:**

This study shows that while allergen-induced lung-homing of endothelial progenitor cells, increased tissue vascularization and development lung dysfunction can occur in the absence of eosinophils, the presence of these cells worsens the pathology of the allergic response.

## Introduction

Despite the development of effective anti-inflammatory therapies and improved delivery approaches, airflow obstruction in asthma is often not fully reversible, and many asthmatics experience an accelerated and progressive loss of lung function over time. Histopathological studies of resected lung and bronchial biopsies have demonstrated several stereotypic changes that correlated with asthma severity [1] suggesting that remodelling of the airway wall may have profound functional consequences, perhaps even greater than those associated with inflammation [2] [3].

Angiogenesis (formation of new blood vessels) in the bronchial submucosa is one of the most consistent features of the asthmatic lung and has been shown to be associated with disease severity [1], [4]–[7]. However, despite an increasing interest, the contribution of the microvascular bed to airway remodelling in asthma is not fully elucidated [8]. It is proposed that increased vascularity assessed as increased vessel number and increased vessel engorgement, can directly increase airway wall thickness causing airway luminal narrowing and facilitate inflammatory cell trafficking thus contributing to airflow obstruction and development of airway hyperresponiveness (AHR) [9], [10]. Investigating the biological processes by which angiogenesis occurs in asthma may provide novel therapeutic targets for the treatment of asthma pathology and understanding its contribution to the development of difficult to control symptoms such as the progressive decline in lung function.

It has been proposed that post natal angiogenesis, is a complex process whereby new blood vessels sprouting from extant microvasculature can arise either from the proliferation of resident mature vascular endothelial cells and/or as a result of the lung-homing of endothelial progenitor cells (EPC) from the BM [11]–[13]. The literature indicates that EPC have the potential to produce growth factors that stimulate local angiogenic responses in a paracrine fashion or incorporate into existing microvessels thus acting as building blocks to form new vasculature [4], [12], [14]. Regardless of the exact role of these progenitor cells, increased mobilization of EPC have been detected in several inflammatory lung conditions including atopic asthma [15]. In asthmatics, EPC numbers are increased in number in the peripheral blood and demonstrate a more proliferative phenotype with the potential to form more tube-like capillary structures in culture, compared to normal non-atopic subjects [15]. More recently, we have shown that following allergen inhalation challenge in asthmatics who develop airway eosinophilia and delayed AHR greater numbers of sputum-extracted EPCs and increased vessel numbers and size in endo-bronchial biopsies were detected [16]. Although studies have reported that CXCR2 ligands promote lung-homing of EPC [17] and that IL-25 and thymic stromal lymphopoietin (TSLP) may promote angiogenic responses [18] [19], mechanisms that orchestrate lung accumulation of EPC in allergic asthma have not been fully investigated.

Using both acute- and chronic- Ag (Ag) exposure protocols, a rapid and sustained increase in microvessel density (MVD) within the lungs of BALB/c mice has been reported [15]. These changes correlated directly and closely with increased airway responsiveness and EPC lung accumulation. In further studies, using the fact that stromal cell derived factor-1 (SDF-1) is a potent progenitor cell chemoattractant, we treated mice intranasally (i.n.) with a CXCR4 antagonist, AMD3100, which blocks SDF-1 mediated effects [20]. This treatment inhibited Ag-induced EPC lung-homing, vascularization, airway eosinophilia and development of AHR. Since SDF-1 is also an eosinophil chemoattractant, and it has been shown that eosinophils produce factors, it remains unclear as to whether AMD3100 acted directly on EPC to attenuate lung accumulation or in-directly through its anti-inflammatory effects on eosinophils. To investigate this issue, we have used a well described transgenic mouse that is devoid of eosinophils (PHIL +/+ mice) [21] and assessed whether Ag-induced lung accumulation of EPC and lung-angiogenesis are observed in these mice compared to wild type (WT) mice.

Our data show that chronic HDM exposure caused a significant increase in lung accumulation of EPC, bronchial vascularity, airway inflammation, and AHR in WT mice and that these Ag-induced changes were significantly greater in WT compared to eosinophil-deficient (PHIL +/+) mice. Localised intranasal delivery of AMD3100 significantly attenuated lung accumulation of all outcome measures in both groups of mice. Our data indicate that EPC recruitment to the lungs in allergic inflammatory responses is mediated by CXCR4/SDF-1 axis acting directly on the progenitor cells. In addition, this study shows that while Ag-induced lung accumulation of EPC and increased lung vascularization *can* occur in the absence of eosinophils, the presence of these cells may have a role in worsening of the pathology of allergic airways disease.

## Materials and Methods

### Ethics Statement

All procedures were reviewed and approved by the Animal Research Ethics Board at McMaster University (Hamilton, ON, Canada). The protocol was approved by the Committee on the Animal Ethics Review Board, McMaster University, Hamilton (Permit number AUP# 10-12-76). All surgery was performed under sodium pentobarbital anesthesia to minimize suffering.

### Animals

Female BALB/c and PHIL eosinophil deficient mice (BALB/c background) were bred and maintained in a pathogen-free environment. PHIL eosinophil-deficient male mice (8^th^ generation BALB/c background; courtesy of Dr. J.J. Lee) were mated with WT BALB/c females (8 week old; Charles River Laboratories, Ottawa, ON). Female PHIL eosinophil-deficient mice (8 week old) and WT littermate controls were kept in a pathogen-free environment.

### Allergen Exposure- Chronic Exposure Protocol

**Figure 1:**
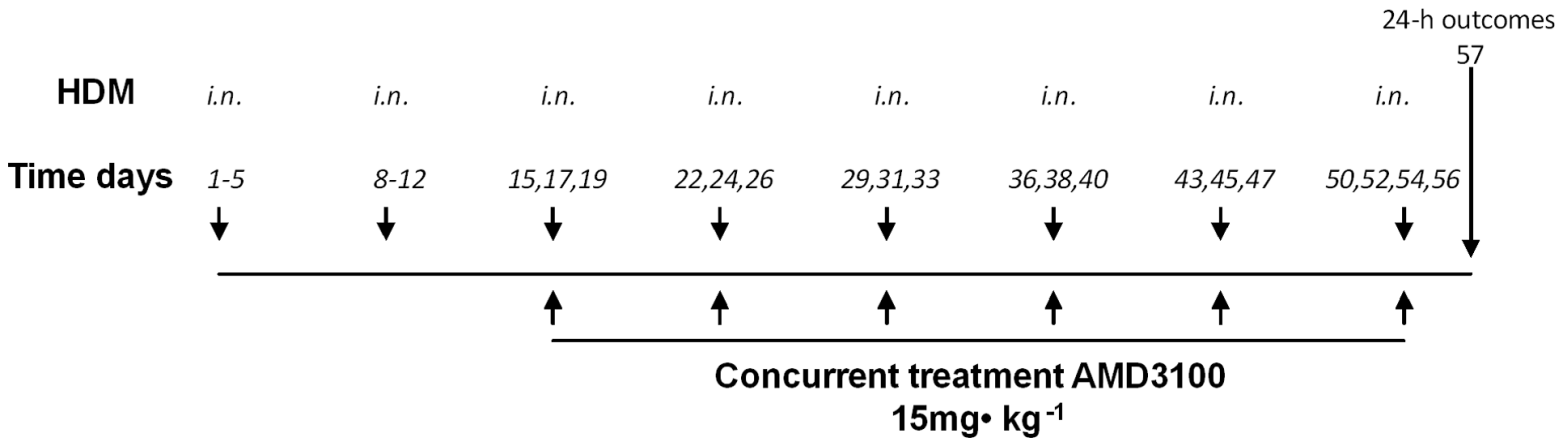
Chronic sensitisation and drug treatment protocol. Wild type (WT) and eosinophil deficient (PHIL +/+) BALB/c mice were sensitized to house dust mite (HDM; 15 µg/µl) or saline (SAL) *i.n.* on days 1–5 and 8–12 and challenged *i.n.* with HDM or SAL 3 days per week for the following 6 weeks. During the challenge phase, treatment groups were administered AMD3100 (15 mg·kg^−1^) or vehicle (*i.n.*) 4 hours prior to HDM or SAL challenge. Outcome measures were made 24 h after the final HDM or SAL challenge (day 57).

Mice (PHIL or WT; n = 10/group) were sensitised and challenged as previously described [22] with modification (Figure 1). Briefly, mice were exposed to HDM *Dermatophagoides pteronyssinus* extract (Greer Laboratories, Lenoir, NC) intranasaly (*i.n.*) (25 µg in 35 µL) for 5 days per week for two weeks, followed by every other day exposures, weekly, for 6 weeks. Outcomes were measured at day 57 (24 h post final exposure).

### Treatment Intervention

Mice were given AMD3100 *i.n.* (Sigma, Mississauga, ON, Canada), a CXCR4 antagonist [23]. Optimal dosing of the drug (15 mg/Kg) was previously established as the lowest dose capable of attenuating allergen-induced increases in airway vascularity in a brief allergen exposure protocol [20]. Concurrent treatment with AMD3100 or saline vehicle was administered 4 h before each allergen challenge during days 15–56 of exposure (**Fig.** **1**).

### Airway Methacholine Responsiveness

Airway responsiveness to intravenous methacholine (MCh) was measured based on the response of airway resistance using the FlexiVent ventilator system (SCIREQ, Montreal, QC, Canada), as previously described [22]. Maximal airway resistance was calculated in response to up to 25 mg/mL methacholine *i.n.* (n = 10 per group).

### Bronchoalveolar Lavage

Bronchoalveolar lavage (BAL) and differential cell counts were performed as previously described in detail [24].

### Progenitor Cell Isolation, Immunostaining and Flow Cytometry

Mice were sacrificed by exsanguination *via* cardiac puncture and lungs were perfused clear of blood with saline and removed from the thoracic cavity, as previously described [25]. Lung tissue-associated cells were extracted from the right lung by mincing and enzymatic digestion, as previously described [20]. Mononuclear cells were collected following density gradient centrifugation (400 *g* for 20 min) over Histopaque (Sigma, Oakville, Ontario, Canada).

Cells were immunostained with Sca-1-FITC, c-kit-PE (BD Bioscience, Oakville, ON, Canada) and VEGFR2-APC (eBioscience Inc., San Diego, CA, USA), or isotype control antibodies (40 min at 4°C), and fixed in PBS with 1% paraformaldehyde (BDH Laboratory Supplies, Mississauga, ON, Canada), and cell data (100,000 events in the lymphomononuclear region) were acquired using a FACSCaliber flow cytometer equipped with a 488-nm argon ion laser (BD Instrument Systems, Mississauga, ON, Canada). Primitive progenitor cells (Sca-1^+^c-kit^+^) and lineage-committed EPCs (Sca-1^+^c-kit^+^VEGFR2^+^) were enumerated using the Cellquest software package (BD Biosciences). The flow cytometric gating strategy are previously described in detail in [15]. Doyle et al., 2011 [20]. Absolute numbers of cells were calculated using the percentage of population positivity obtained by flow cytometery and the total white cell count.

### Lung Histology and Morphometry

The left lung was perfused with saline, formalin fixed, embedded in paraffin and cut into 3-µm sections. Bronchial vascularity was identified by staining with polyclonal rabbit anti-human von Willebrand factor (vWF) (Dako, Carpinteria, CA, USA), which cross-reacts with the mouse antigen [26]. Slides were analysed using a customised digital image analysis system (Northern Eclipse; Empix Imaging, Mississauga, ON, Canada). The main airway in the tissue section was identified and traced, allowing the program to only identify the area within a 50 µm bandwidth from the airway. Only vessels that were vWF+e within this area and less than 10 µm in diameter were included in vessel quantification. Microvessel density (MVD) was then calculated by dividing the total positive vessels by the bandwidth areaFixed lung sections (3 µm) were also stained with hematoxylin and eosin (H&E) to identify tissue eosinophils. Eosinophils within a 50-µm bandwidth from the main airway were enumerated and calculated as total number of eosinophils per bandwidth area using Northern Eclipse software.

### Cytokine/Chemokine Assessments

Lung tissue supernatants were analyzed by ELISA, vascular endothelial growth factor (VEGF) and SDF-1, TSLP, IL-25 and IL-33 kits (R&D Systems, Minneapolis, MN, USA). All data were normalized for lung weight.

### Statistical Analysis

Data are presented as mean ± SEM. Analysis was performed using STATSISTICA software (Statsoft INC., Tulsa, OK, USA) to perform ANOVAs; *post hoc* analyses for between groups comparisons were performed using Duncan's test. Alpha was set at 0.05.

## Results

### Progenitor Cells: HDM exposure stimulated progenitor cell lung accumulation which was inhibited by treatment with AMD3100 in WT and PHIL mice

**Figure 2:**
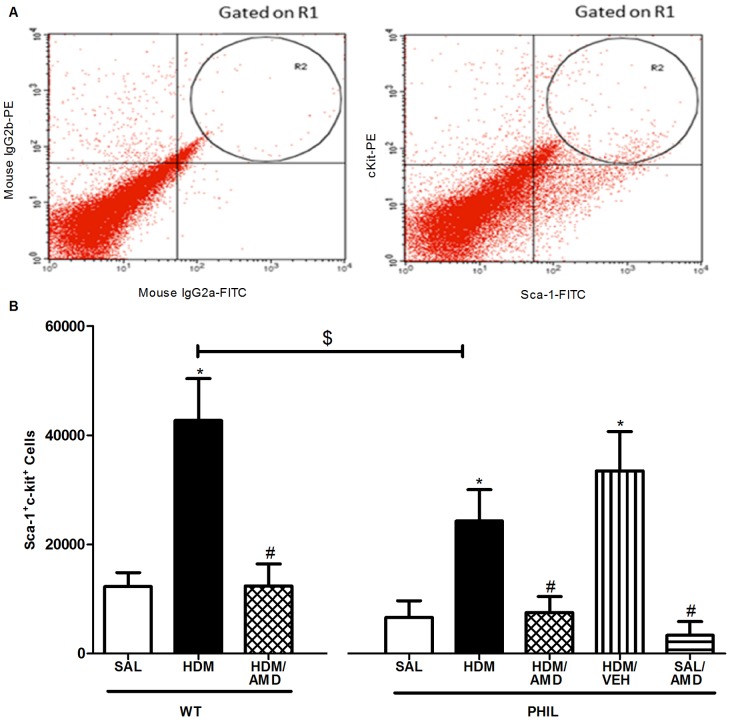
Flow cytometric enumeration of lung-extracted primitive progenitor cells. (A) On a dot plot of linear side scatter vs Sca-1 FITC plot, a region R1 was drawn to determine Sca-1+ cells lymphomononuclear cells. From this region events were gated on a dot plot of Sca-1+/C-kit+ cells as shown in Figure panel A. Compared to isotype controls, primitive progenitors were identified as identified as Sca-1+c-kit+ cells. (B) Lung extracted cells were harvested 24 h after final i.n. challenge with saline (SAL), house dust mite (HDM) or HDM+AMD3100 (n = 10 per group). In WT and PHIL mice, a significant increase in Sca-1+c-kit+ cells was detected in HDM compared to SAL which was attenuated following treatment with AMD3100. There were significantly lower levels of primitive progenitors in PHIL compared to WT mice. *p<0.05 compared with SAL; #p<0.05 compared with HDM; $p<0.05 compared with WT HDM. Data expressed as mean ± SEM.

**Figure 3:**
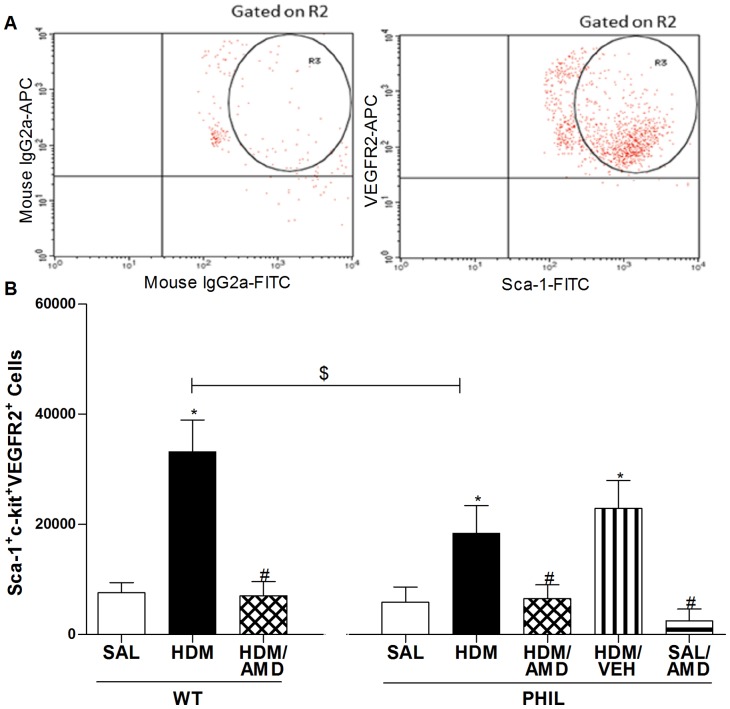
Flow cytometric enumeration of lung-extracted vascular endothelial progenitor cell. (A) Compared to isotype controls, (B) Lung extracted cells were harvested 24 h after final i.n. challenge with saline (SAL), house dust mite (HDM) or HDM+AMD3100 (n = 10 per group). Compared with SAL, a significant increase in EPC was detected in HDM groups in WT and PHIL mice and this was attenuated by AMD3100. *p<0.05 compared with SAL; #p<0.05 compared with HDM; $p<0.05 compared with WT HDM. Data expressed as mean ± SEM.

Using a chronic allergen exposure model in WT and PHIL BALB/c mice, primitive progenitor cells (PC; Sca-1^+^c-kit^+^) and endothelial progenitors (EPC; Sca-1^+^c-kit^+^VEGF2R^+^) were enumerated in lung-extracted tissue, 24 h post-challenge with allergen (HDM) or saline (SAL) (Fig. 2&3). Compared to SAL, PC levels were significantly increased following HDM exposure in WT and PHIL mice (Fig. 2). Similarly, compared to SAL, EPC levels were significantly increased following HDM exposure in WT and PHIL mice (Fig. 3). Between group comparisons showed that PHIL/HDM exposed mice had a significantly lower numbers of lung extracted PC (Fig. 2) and EPC (Fig. 3). Intranasal administration of AMD3100 given concurrently with HDM exposure significantly attenuated lung PC (Fig. 2) and EPC numbers (Fig. 3) to SAL levels in both groups of mice. In control experiments performed in PHIL mice, we found there was no difference between HDM treated group vs HDM/vehicle group which supported a specific effect of the drug (Fig. 2&3). In addition, there was no difference in the SAL vs. SAL/AMD treated group indicating that AMD3100 acted to specifically inhibit HDM-induced accumulation of progenitor cells in the lungs.

### Angiogenesis: HDM exposure increased bronchial vascularity which was attenuated by treatment with AMD3100 in WT and PHIL mice

**Figure 4:**
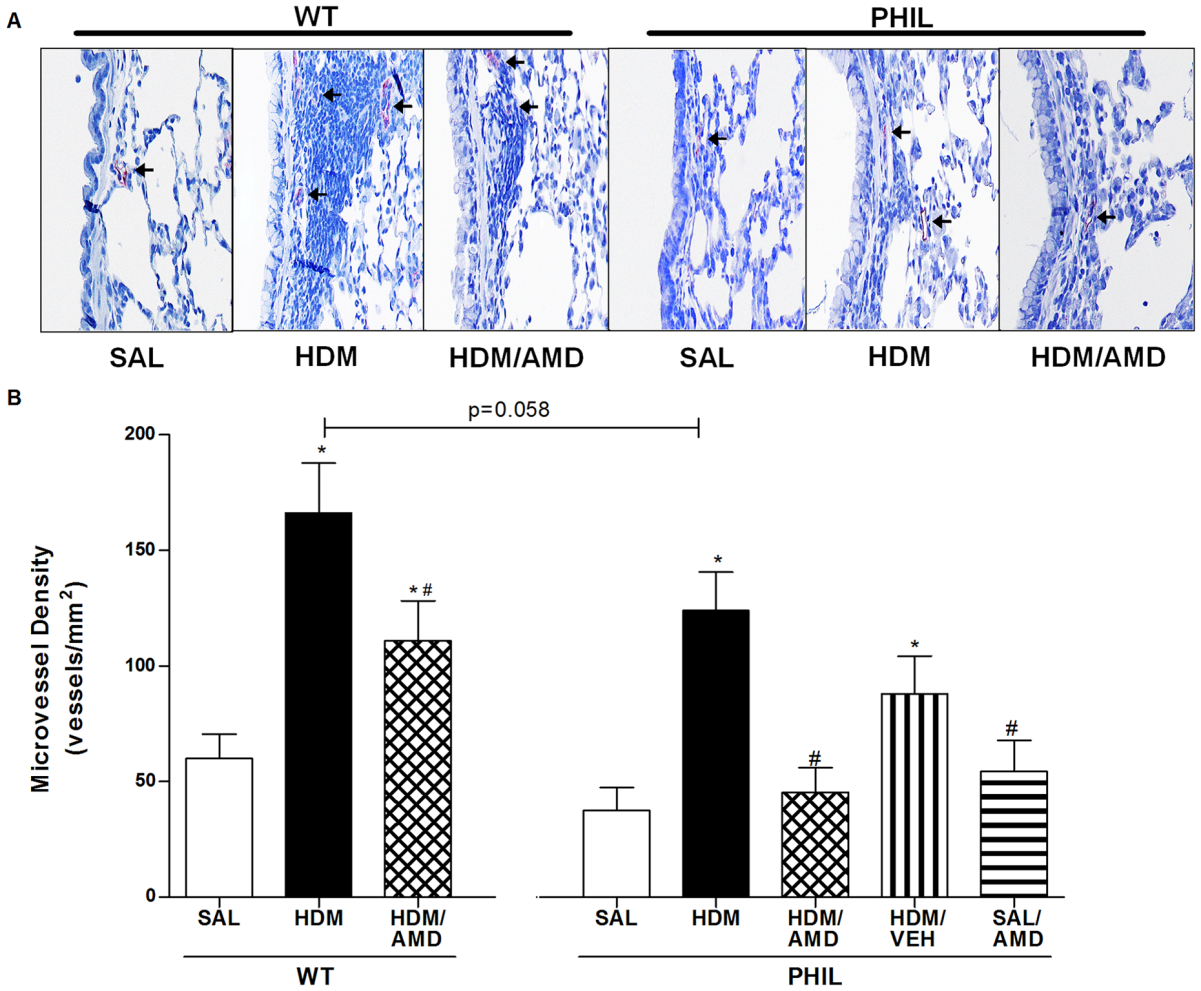
Microvessel density (MVD) assessed by immunostaining for von Willebrand factor in Lung tissue sections. (A) Images from 24 hrs post-final allergen challenge at 40X magnification. Enumerated vessels were 10 µm in diameter or less (arrows). Scale bars = 50 µm (n = 10 per group) (B) MVD levels increased significantly in HDM compared with SAL groups in both WT and PHIL mice which was attenuated by AMD3100. Between group comparisons showed a trend for lower MVD levels in PHIL versus WT mice (p = 0.058). *p<0.05 compared with SAL; #p<0.05 compared with HDM. Data are expressed as mean ± SEM.

Lung vascularity was assessed by staining for vWF in mouse lung slices and enumerating microvessel density (MVD) (Fig. 4). MVD was significantly increased in HDM when compared to SAL groups for both WT and PHIL mice (Fig. 4). In WT mice, compared to the HDM group treatment with AMD3100 (HDM/AMD group) significantly attenuated MVD levels although these levels remained significantly greater than SAL levels. Similarly in PHIL mice, AMD3100 treatment significantly attenuated MVD levels compared to HDM group and these levels were comparable with SAL levels (Fig. 4). A vehicle effect was not observed in PHIL mice supporting a specific effect of AMD3100. Between group comparisons showed a trend towards lower lung vascularity in PHIL/HDM mice compared to WT/HDM group (p = 0.058) (Fig. 4).

### Airway Inflammation: Eosinophilia in WT mice was significantly attenuated in lung tissue when allergen-exposed mice were treated with AMD3100

**Figure 5:**
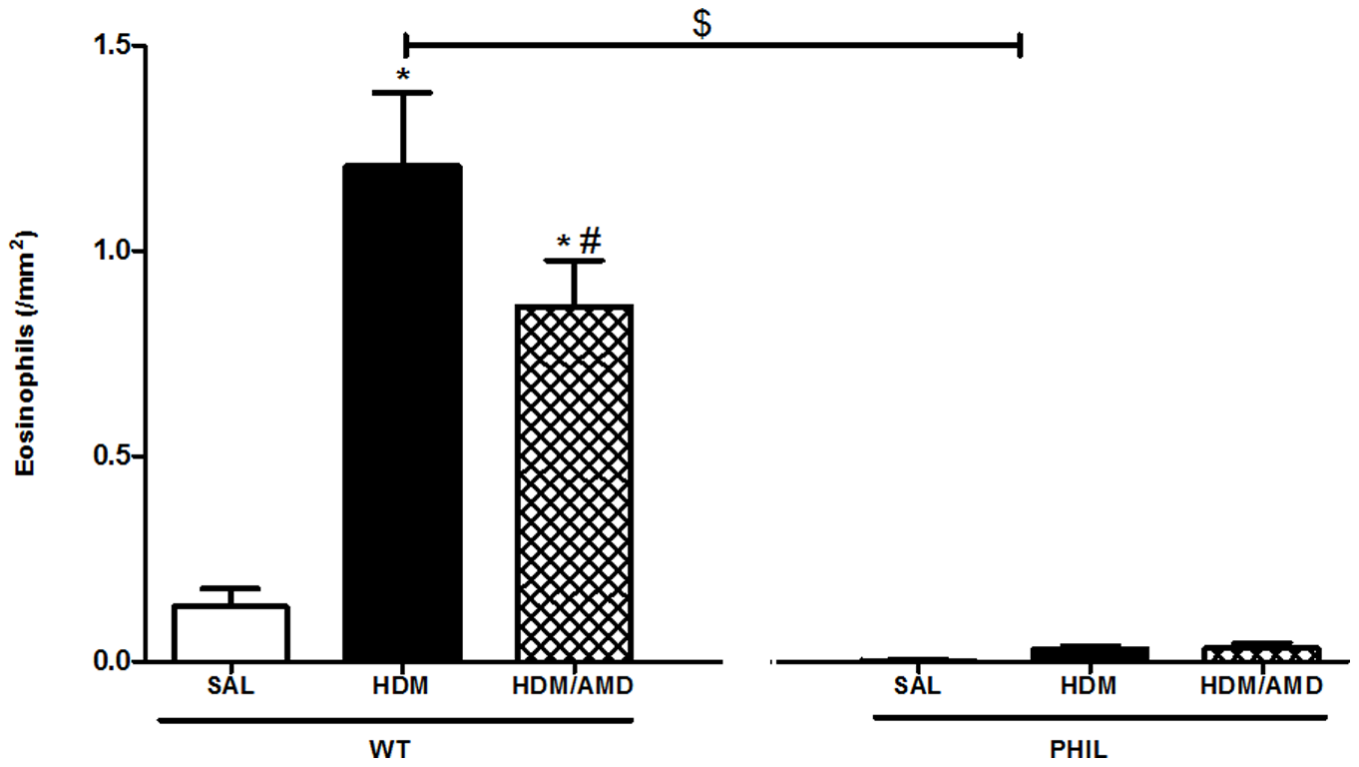
Airway eosinophilia assessed by hematoxylin and eosin stain. In WT mice, house dust mite (HDM) exposure significantly increased eosinophils compared with saline (SAL). This was significantly attenuated by treatment with AMD3100. PHIL mice had negligible numbers of eosinophils. *p<0.05 compared with saline; #p<0.05 compared with HDM. Data are expressed as mean ± SEM. (n = 10 mice per group).

To assess for tissue eosinophilia, lung sections were stained with hematoxylin and eosin. In WT mice, there was a significant increase in eosinophils in the HDM group compared to SAL and these levels were attenuated in the AMD3100 treated group, though not to SAL levels (Fig. 5). As expected, PHIL mice had negligible tissue eosinophils in all groups (Fig. 5).

To assess anti-inflammatory effects of AMD3100 on other pro-inflammatory cells, differential cell counts were performed on BAL samples. In WT mice, compared to SAL, there was a significant increase in the total cell count including eosinophils, neutrophils and lymphocytes in WT/HDM group (Table 1). Treatment with AMD3100 did not significantly diminish the HDM-induced inflammatory response in WT mice. Similar findings were also observed in PHIL mice (Table 1).

**Table 1:** Airway inflammation as measured by bronchial alveolar lavage (BAL).

	WT			PHIL		
	SAL	HDM	HDM+AMD3100	SAL	HDM	HDM+AMD3100
**TCC ×10^4^**	3.37±0.54	9.20±1.60*	6.87±0.77*	4.13±0.48	6.83±0.51*	5.69±0.28*
**Eosinophils**	0.01±0.01	2.85±0.47*	2.05±0.14*	0±0	0.02±0.01	0.00±0.00
**Neutrophils**	0.19±0.06	1.15±0.32*	1.35±0.16*	0.18±0.04	1.33±0.31*	1.24±0.14*
**Macrophages**	2.65±0.53	2.87±0.46	1.66±0.20	3.37±0.43	3.03±0.27	2.52±0.16
**Lymphocytes**	0.52±0.06	2.36±0.59*	1.80±0.27*	0.59±0.13	2.46±0.29*	1.92±0.30*

Data are presented as mean ± SEM. TCC: total cell count. Cells were counted in bronchoalveolar lavage samples collected from WT and PHIL mice (n = 10 per group) sensitized by chronic exposure protocol with concurrent treatment with AMD3100. Measurements were made at 24 h after saline (SAL), house dust mite (HDM), or HDM+AMD3100 challenge. *p<0.05 compared to SAL. Data are expressed as mean ± SEM.

### Airway Responsiveness: HDM exposure increased airway sensitivity to MCh which was attenuated by treatment with AMD3100 in WT and PHIL mice

**Figure 6:**
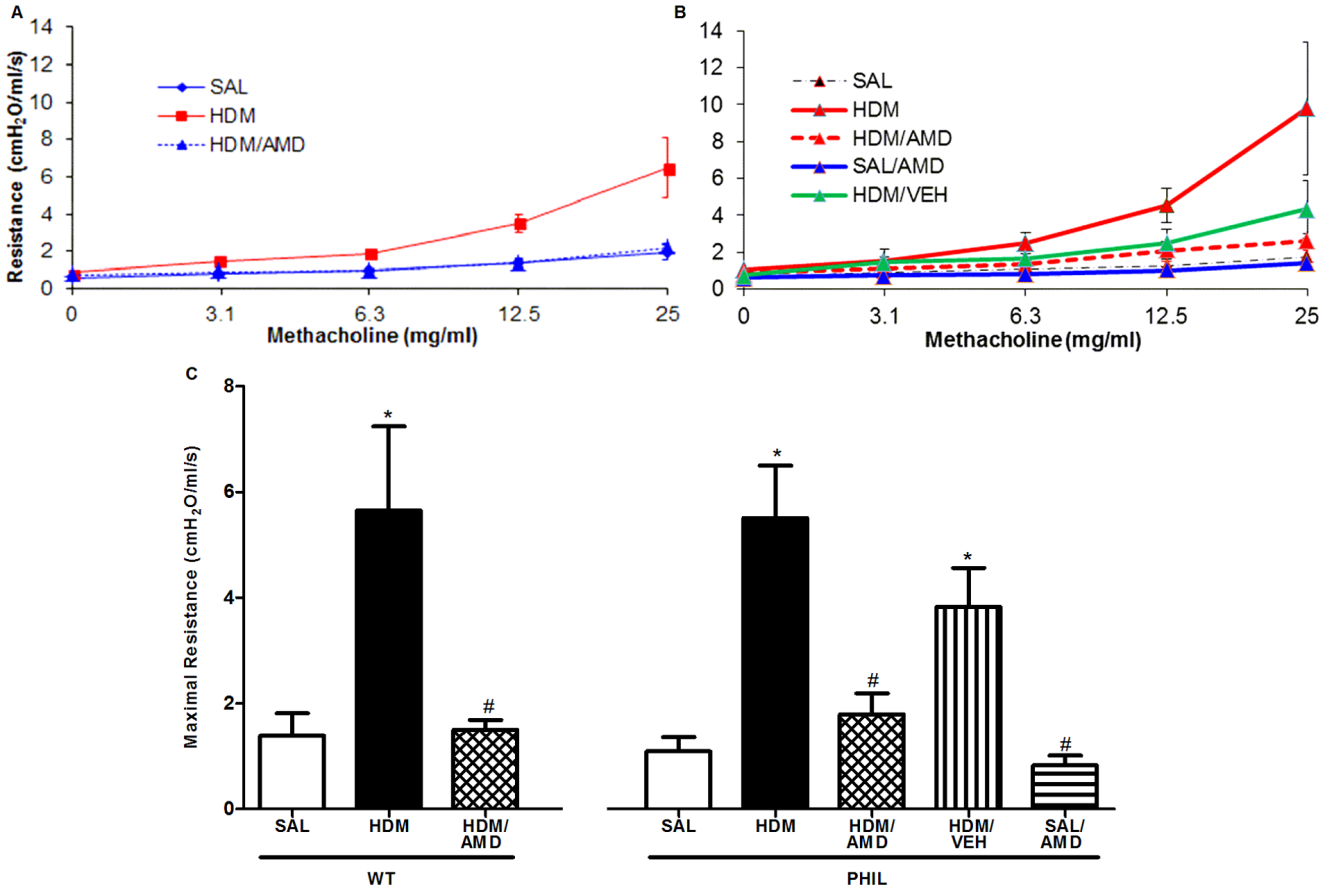
Airway responsiveness as measured by resistance to intranasal methacholine. Airway resistance curves to methacholine for (A) WT and (B) PHIL mice; (C) Maximal airway resistance in response to 25 mg/mL methacholine *i.n.* (n = 10 per group). Data are presented as the maximal resistance between 3.1–25 mg/mL minus the baseline value (0 mg/ml) for each mouse. In WT and PHIL mice, there was a significant increase in methacholine airway responsiveness in HDM versus SAL in WT and PHIL mice. This was significantly attenuated by treatment with AMD3100. Data are expressed as mean ± SEM. *p<0.05 compared with SAL; #p<0.05 compared with HDM.

Airway responsiveness was assessed by measuring the maximum airway resistance to incremental doses of MCh *i.n*. in WT and PHIL mice (Fig. 6A&B). A significant increase in maximum resistance to MCh was observed 24 hrs post-allergen in HDM exposed mice compared to the SAL in both WT and PHIL mice and treatment with AMD3100 significantly attenuated this to SAL levels in both groups of mice (Fig. 6C). Although there was a trend for lower airway resistance in PHIL mice compared to WT mice, between group comparisons, showed no significant difference between these two groups (WT/HDM vs PHIL/HDM mice) (Fig. 6C).

### Levels of Tissue Associated Cytokines and Pro-angiogenic factors

Tissue Associated levels of VEGF TSLP and IL-33 were determined in supernatants collected from digested lung tissue (n = 10 mice per group) by ELISA in samples collected 24 h post-challenge from all the various treatment groups (Table 2). In both WT and PHIL mice, a significant increase in VEGF levels were detected in HDM compared to SAL group 24 h post Ag- challenge and drug treatment had no effect on these levels. In addition, there were no between group differences observed. TSLP was found to increase significantly in the HDM compared to SAL groups for both WT and PHIL mice and treatment with AMD3100 had no effect on these levels. IL-33 levels were not found to be different in the HDM group compared to SAL for both WT and PHIL mice.

**Table 2:** Lung Tissue Associate Levels of Pro-Angiogenic Cytokines and Chemokines.

	WT			PHIL		
Factor Levels (pg/ml)	SAL	HDM	HDM+AMD3100	SAL	HDM	HDM+AMD3100
**VEGF**	1740±190	3460±420*	3660±230*	1850±170	2730±220*	2430±340*
**TSLP**	54.7±2.1	158±12.7*	149±12.6*	48.7±3.06	175±17.6*	149±13.07*
**IL-33**	27.6±1.2	30±2.3	26±2.3	25.3±1.68	36±3.4	35±3.3

Tissue Associated levels of Vascular endothelial derived growth factor (VEGF), TSLP and IL-33 were determined in supernatants collected from digested lung tissue (n = 10 mice per group) by ELISA in samples collected 24 h post-challenge. In WT and PHIL mice, there is a significant increase in levels of VEGF and TSLP that were not attenuated by treatment with AMD3100 in both groups of mice. Compared to SAL, there was no allergen-induced production of IL-33 in either WT or PHIL mice. *: p<0.05 compared with saline (SAL); Data are presented as mean ± SEM.

## Discussion

Asthma has a systemic component involving the mobilisation and lung homing of BM-derived progenitor cells that may contribute to inflammation and tissue remodelling, including angiogenesis [27], [28]. Bone marrow-derived EPCs have been shown to contribute to increased bronchial vascularization through *in situ* differentiation into blood vessels [29] as well as release of proangiogenic factors [30]. Using an ovalbumin challenge model, we have previously shown that inhibiting endothelial progenitor cell accumulation in the lung prevented angiogenic responses and the development of AHR [20]. However, the SDF-1α/CXCR4 axis within the lung, that stimulates the homing of progenitor cells, is also an eosinophil chemoattractant [31] and our study showed that treatment with AMD3100 inhibited allergen-induced airway eosinophilia [20]. It was therefore unclear whether AMD3100 acted directly on EPCs to attenuate lung accumulation or indirectly through its anti-inflammatory effects on eosinophils. The current study tested the hypothesize that lung angiogenesis is an early tissue remodeling event that is independent of eosinophilic inflammation and that mobilization, lung-homing and *in-situ* differentiation or activation of vascular endothelial progenitor cells is a major component of this response.

Using house dust mite, an aeroallergen that is more physiologically relevant to human asthma, this study showed that 1) chronic allergen exposure induces EPC lung-homing, increased bronchial vascularity and development of lung dysfunction in WT BALB/c mice; 2) Similar effects were observed in HDM exposed PHIL mice albeit at lower levels than WT mice suggesting that while the initiation of allergen-induced effects may not be dependent upon eosinophils, the recruitment and activation of eosinophils worsens the pathology of the inflammatory response; 3) Treatment with AMD3100 was comparable in the presence or absence of eosinophils (i.e in WT and PHIL mice) demonstrating that the SDF-1-CXCR4 axis acts directly on EPC to stimulate lung-homing and promote increased bronchial vascularity and development of airway dysfunction in response to allergen exposure.

As in humans, the murine lung has two blood supplies, the pulmonary and bronchial systems. The functional role of the pulmonary circulation is related to gas exchange while the bronchial circulation provides nutrients and inflammatory cells to the peripheral lung via oxygenated blood from the systemic circulation. Gross anatomic studies have long demonstrated bronchial arteries supplying bronchial and structural tissues of the murine lung. The concept of the bronchial arteries also supplying the peripheral lung in mice has been suggested by the presence of lung structure in the presence of pulmonary artery obstruction [32]. A study by Ravnic et al., detailed murine bronchopulmonary microcirculation and provided morphological evidence for (i) peripheral bronchial circulation, (ii) interconnections between bronchial and pulmonary circulation in the distal bronchial arteries and at the level of the alveolar capillaries both arising at vessels <20 µm diameter, and (iii) functional evidence of increased bronchial perfusion to alveolar capillaries during a peripheral mononuclear inflammatory response to intratracheal installation of peptide-hapten trinitrophenol demonstrating an adaptive role of the bronchial circulation in pulmonary inflammation [33]. The latter findings are consistent with clinical findings of bronchial hyperplasia in chronic inflammatory disorders.

In the current study we stained newly formed microvessels with vWf which is a glycoprotein that is essential for blood coagulation and vessel wall repair that is stored in Weibel-Palade bodies within the cytoplasm of endothelial cells [34]. Comparative immunohistochemical staining and confocal laser microscopy of vWf throughout varying calibers of pulmonary vasculature have shown that vWf is a consistent positive marker for all vessels greater than 10 µm and is undetectable in alveolar capillary endothelial cells, but is highly expressed in venules [32], [35]. These vessels sizes support our own determination of newly formed microvessels within a 50 µm bandwidth from the airway and ≤10 µm in diameter. By this method we have shown that there is a significant increase in bronchial vascularity in murine lung following HDM challenge in sensitized WT and PHIL mice. In addition, we and others have previously demonstrated a rapid and sustained allergen-induced lung homing of EPCs which correlated with increased bronchial vascularity and airway resistance following ovalbumin exposure in sensitized WT mice [15], [20]. In addition, kinetic studies showed the EPC recruitment preceded the onset of lung vascularization and airway eosinophilia [15]. Further studies in this mouse model showed that lung-extracted EPC expressed eotaxin which could in-turn recruited eosinophils to the site of inflammation [36]. More recently, nascent epithelial cells have been shown to promote Th2 responses suggesting that EPCs homing to the lung in response to allergen challenge may act as early initiators of allergic inflammatory responses [37]. The current study shows for the first time that HDM-exposure induced increases in lung-extracted EPC and bronchial vascularity 24h post-Ag challenge in both WT and eosinophil deficient PHIL mice. Comparable changes in airway resistance are consistent with previous studies [38]. This suggests that allergen exposure in PHIL mice results in lung remodelling changes even in the absence of eosinophils albeit at lower levels compared to WT mice. Thus although eosinophils are not necessary for the onset of airway disease, they do appear to contribute to worsening the lung remodelling changes in WT mice. This is in line with previous findings that eosinophil-derived granule proteins and cell derived factors (angiogenin, VEGF, and NGF) have pro-angiogenic effects and can stimulate lung vascularization [39]–[41].

In this study, AMD3100 was administered *i.n.* during the chronic phase of the HDM exposure model so as to localize the drug effects to the lung. Assessment of the lung tissue showed that drug treatment attenuated allergen-induced lung EPC levels in WT mice and PHIL mice to Saline levels. Similarly, AMD3100 significantly attenuated MVD levels in PHIL mice to saline levels indicating that increased accumulation of EPC may directly form tube-like structures or promote local angiogenic responses by stimulating proliferation of existing vascular endothelial cells in response to allergen challenge in the absence of eosinophils [42] [43] [28]. By comparison in WT mice, MVD levels were significantly increased compared to SAL levels in HDM group, and treatment with AMD3100 attenuated these numbers although not to saline levels (Fig. 4). Since HDM-induced eosinophil numbers in the peribronchial region were not completely abrogated by treatment with AMD3100 (Fig. 5), it is likely that eosinophil-derived products may also play a role in promoting the local vascularization of the lungs. This is supported by the fact that vascularization is attenuated to saline levels in the PHIL mice that lack eosinophils but not in WT mice (Fig. 4).

In the current study, BAL eosinophilia in WT mice treated with the drug, AMD3100 remained elevated (Table 1), contrary to the reduction of airway eosinophilia reported in our previous OVA-challenge study [20], [39]. Differences in the effect of the drug on Ag-induced airway eosinophilia (taken from BAL fluid) between OVA and HDM models maybe be caused by the fact that HDM exposed mice develop a more robust eosinophila that is not as easily attenuated as that seen in OVA-exposed mice. In a study comparing OVA and HDM chronic exposure models in BALB/c mice, the magnitude of the airway eosinophilia was found to be approximately 3 times greater in HDM mice 24hours post allergen exposure [44]. The robust eosinophilia seen in HDM exposed mice may be caused by HDM activating the airway epithelium through the innate immune pathway in addition to activation of the adaptive immune response. It has been shown that HDM-associated ligands (such as proteases Der p 2,7 and endotoxin) are recognize by TLR-4 (toll-like receptor 4) which can stimulate airway epithelial cells to produce TSLP, IL-25, and IL-33. These TLR-4 products further promote inflammatory cell (i.e. eosinophil) accumulation into the lungs, activate dendritic cells and promote expansion of T_H_2 cells. Through a feedback loop, Th2 cytokines such as IL-13 activate epithelial cell production of cytokines to produce eotaxin, a potent eosinophil chemoattractant [45]. Thus, activation of both innate and adaptive immune pathways may stimulate a greater recruitment force for increased lung-homing of eosinophils compared to ovalbumin challenge.

The role of epithelial-derived cytokines in promoting EPC migration and angiogenic response sin the lungs has been reported. In this study we show that following HDM challenge there is a significant increase in TSLP production in the WT mice and PHIL mice. Treatment with AMD3100 did not affect the production of this cytokine, indicating the overriding effect of the SFDF-1/CXCR4 axis in the lung-homing of EPC in allergic inflammatory responses.

In summary, we report that modulating EPC traffic into the lung attenuated angiogenesis and the development of AHR. Our data indicate that EPC recruitment to the lungs in allergic inflammatory responses is mediated by CXCR4/SDF-1 axis acting directly on the progenitor cells. In addition, we have shown that while Ag-induced lung accumulation of EPC and increased lung vascularization *can* occur in the absence of eosinophils as seen in PHIL mice, the presence of these cells may have a role in worsening of the pathology of allergic airways disease.

## Supporting Information

Data S1 **Data and Stats.**
(XLSX)
